# Exosomes derived from human CD34^+^ stem cells transfected with miR-26a prevent glucocorticoid-induced osteonecrosis of the femoral head by promoting angiogenesis and osteogenesis

**DOI:** 10.1186/s13287-019-1426-3

**Published:** 2019-11-15

**Authors:** Rongtai Zuo, Lingchi Kong, Mengwei Wang, Wenbo Wang, Jia Xu, Yimin Chai, Junjie Guan, Qinglin Kang

**Affiliations:** Department of Orthopedic Surgery, Shanghai Jiao Tong University Affiliated Sixth People’s Hospital, Shanghai, China

**Keywords:** Osteonecrosis of the femoral head, Glucocorticoids, CD34^+^ stem cells, Exosomes, miR-26a, Angiogenesis, Osteogenesis

## Abstract

**Background:**

Damaged endothelial cells and downregulated osteogenic ability are two key pathogenic mechanisms of glucocorticoid (GC)-induced osteonecrosis of the femoral head (ONFH). Recent studies suggested that transplantation of CD34^+^ stem cell-derived exosomes (CD34^+^-Exos) can treat ischemic diseases by promoting neovascularization and that miR-26a is an important positive regulator of osteogenesis. Moreover, the biological effect of exosomes is closely related to their cargo miRNAs. However, it is not clear whether increasing the abundance of miR-26a in CD34^+^-Exos will inhibit the progress of GC-induced ONFH.

**Methods:**

MiR-26a was overexpressed in CD34^+^-Exos (miR-26a-CD34^+^-Exos) to increase their osteogenic potential. The angiogenic potential of miR-26a-CD34^+^-Exos was then examined through evaluations of migration and tube-forming capacities in vitro. In addition, in order to observe the osteogenic effect of miR-26a-CD34^+^-Exos on bone marrow stromal cells (BMSCs), Alizarin red staining, alkaline phosphatase (ALP) activity assays, and qPCR were carried out. Finally, miR-26a-CD34^+^-Exos were injected into a GC-induced ONFH rat model to prevent the progress of GC-induced ONFH. The biological effects of miR-26a-CD34^+^-Exos on the ONFH model were evaluated by micro-CT, angiography, and histological staining.

**Results:**

Our data showed that miR-26a-CD34^+^-Exos enhanced human umbilical vein endothelial cell migration and tube-forming capacities. Furthermore, miR-26a-CD34^+^-Exos strengthened the osteogenic differentiation of BMSCs under the influence of GCs in vitro. Finally, the miR-26a-CD34^+^-Exos increased the vessel density and trabecular bone integrity of the femoral head in the GC-induced ONFH rat model, which inhibited the progress of ONFH.

**Conclusions:**

MiR-26a-CD34^+^-Exos protect the femoral head from damage caused by GCs by strengthening angiogenesis and osteogenesis. The biological effect of miR-26a-CD34^+^-Exos make them suitable for application in the prevention of GC-induced ONFH.

## Introduction

Glucocorticoid (GC)-induced osteonecrosis is one of the most common causes of osteonecrosis of the femoral head (ONFH) [1]. Although related pathogenic hypotheses have been discussed, the exact pathogenesis of GC-induced ONFH remains unknown. According to previous research, the pathogenesis of GC-induced ONFH could be summarized as the following two aspects: (i) damaged blood supply to the femoral head and (ii) weakened osteogenic activity [2–6].

The overuse of GCs can cause damage and dysfunction to vascular endothelial cells [2, 7]. Several studies have shown that GCs can directly impair endothelial cells, leading to a hypercoagulable state and abnormal microthrombus formation in the necrotic region of the femoral head, which severely reduces the blood supply to the trabecular bone [8, 9]. Meanwhile, osteogenesis is also inhibited by excessive GC treatment [4]. GCs are reported to be associated with a reduction in bone mineral density and the breakdown of bone trabeculae [10]. In addition, GCs downregulate the expression of Runt-related transcription factor 2 (RUNX2) and alkaline phosphatase (ALP), which suppress osteogenesis in vitro and bone formation in vivo [10–12]. Therefore, enhancing angiogenesis and osteogenesis simultaneously is the key to the prevention or early treatment of GC-induced ONFH.

Exosomes (Exos) are microvesicles with a diameter of 40–150 nm formed by a continuous process of endocytosis-fusion-excretion, which have aroused extensive attention [13]. Exos have a very similar membrane to their parent cells and contain analogous bioactive factors including cytokines, growth factors, lipids, and non-coding RNAs [14]. Once Exos enter the cell, they can mediate cell-to-cell signal transduction and exert biological regulatory effects. The use of Exos in the treatment of ONFH has been reported previously, but the treatment efficacy of Exos still needs further enhancement for clinical application [15, 16]. Interestingly, the composition of the Exos can be modified to adjust their biological function [17]. To better apply Exos in the treatment of ONFH, the Exos should have simultaneous potential for both angiogenesis and osteogenesis.

CD34^+^ stem cells are a group of vessel progenitor cells, which have extraordinary angiogenic properties and have been used to treat limb ischemia in the clinic [18, 19]. Studies have reported that intramuscular injection of autologous CD34^+^ stem cells can increase angiogenic activity and myogenesis in critical limb ischemia and decrease the amputation rate [18, 19], which is promising for the treatment of ischemic disease. However, stem cell transplantation still has many defects, such as the low survival rate, genetic variation, and tumorigenesis, which limit the application of CD34^+^ stem cells in ischemic disease [16, 20].

With these concerns, Exos from CD34^+^ stem cells (CD34^+^-Exos) were developed as a superior choice to promote neovascularization in the treatment of ischemic disorders. Researchers found that CD34^+^-Exos could strengthen tube formation of endothelial cells in vitro and angiogenesis in vivo [21]. Prabhu et al. demonstrated that CD34^+^-Exos promote vascular angiogenesis in a mouse hindlimb ischemia model [22]. However, the effect of CD34^+^-Exos in the prevention or early treatment of GC-induced ONFH remains unknown.

MiRNAs are endogenous non-coding RNAs and some miRNAs play an important role in osteogenic differentiation. It is reported that miR-26a increases the expression of ALP, collagen I (COL I), osteocalcin (OCN), and bone morphogenetic protein-2 (BMP-2) in adipose-derived stem cells (ADSCs) and promotes their collagen secretion and mineralization [23]. In bone marrow stromal cells (BMSCs), miR-26a can also upregulate the expression of ALP and OCN and promote their osteogenic differentiation in vitro and in vivo [24]. In addition, many researches have shown that miR-26a could promote the process of osteogenesis by regulating multiple osteogenic factors such as Wnt, Smad1, or GSK-3β signal pathway [25, 26]. Therefore, we upregulated the content of miR-26a in CD34^+^-Exos (miR-26a-CD34^+^-Exos) to increase their osteogenic potential. However, the influence of miR-26a on angiogenesis has also been studied. Moreover, the effects and mechanisms of miR-26a on angiogenesis are diverse in different diseases [27–30]. It is unclear what effect miR-26a has on angiogenesis in GC-induced ONFH.

In the present study, we transfected miR-26a into CD34^+^ stem cells. The Exos were extracted from transfected cells, and the angiogenic potential of miR-26a-CD34^+^-Exos was examined in vitro. Next, the osteogenic potential of miR-26a-CD34^+^-Exos was also analyzed in vitro. Finally, the miR-26a-CD34^+^-Exos were injected into a rat model of GC-induced ONFH and the in vivo protective effect of miR-26a-CD34^+^-Exos was examined. The current study not only provides a new method to modify the osteogenic ability of CD34^+^-Exos, but also lays a solid foundation for the use of CD34^+^-Exos in GC-induced ONFH.

## Materials and methods

### Cell culture

Human CD34^+^ stem cells were obtained from HemaCare Corporation (Northridge, CA, USA). The cells were isolated from a G-CSF mobilized leukapheresis product by positive selection using CD34^+^ immunomagnetic bead separation. After isolation, expression of the surface marker CD34 of CD34^+^ stem cells was confirmed by flow cytometry. CD34^+^ stem cells from passages 3 to 9 were cultured in StemSpan™ SFEM II serum-free cell culture medium (Stem Cell, Beijing, China) and StemSpan™ CC100 (Stem Cell), and the serum-free medium was changed every 2–3 days. After the cell confluence over 90%, the StemSpan™ SFEM II conditioned medium was collected for extracting exosomes.

Human BMSCs were purchased from the Cell Bank of the Chinese Academy of Sciences (Shanghai, China). BMSCs were cultured in α-MEM (Hyclone, Logan, UT, USA) containing 10% fetal bovine serum (FBS; Gibco, Carlsbad, CA, USA), 100 U/mL penicillin, and 100 μg/mL streptomycin (Beyotime, Guangzhou, China). Human umbilical vein endothelial cells (HUVECs) were purchased from the Sciencell Corporation (Shanghai, China). HUVECs were maintained in ECM medium (Sciencell) with 5% FBS and 1% ECGS (Sciencell). BMSCs and HUVECs were used from passage 3 to 6 in this study. These three cell types were cultured at 37 °C in an incubator (Thermo Fisher, MA, USA) under 5% CO_2_ in air, and the medium was refreshed every 3 days.

### Lentiviral transfection of miR-26a into CD34^+^ stem cells

Lentiviral transfection particles of miR-26a were obtained from Genechem Corporation (Shanghai, China). Transfection procedures were performed according to standard protocol. Briefly, a suspension of 10^5^ cells/mL was co-incubated with 50 μL virus particles and 20 μL infection solution (15 μL HiTransB-1 and 5 μL HiTransB-2) for 12 h. After 72 h, a fluorescence microscope (Olympus IX 70, Tokyo, Japan) was used to observe transfection efficiency. Quantitative polymerase chain reaction (qPCR) was used to measure the stable expression of miR-26a in miR-26a-CD34^+^-Exos 20 days after of 2 μg/mL puromycin sorting. In addition, cell counting kits (CCK-8, Dojindo, Kumamoto, Japan) were used to test cell viability. Five thousand CD34^+^ stem cells, negative control (NC)-green fluorescent protein (GFP)-transfected CD34^+^ stem cells, or miR-26a-GFP-transfected CD34^+^ stem cells were seeded into each well of a 96-well plate, and 10 μL CCK-8 solution was added into each well after 24 or 72 h of culture. After 2 h incubation, the absorbance was measured using a microplate reader (BioTek, Winooski, VT, USA) at 450 nm. The optical density (OD) values represent the viability of cells.

### Isolation of exosomes

Exosomes were isolated from the serum-free medium of the different culture groups (CD34^+^ stem cells, NC-GFP-transfected CD34^+^ stem cells or miR-26a-GFP-transfected CD34^+^ stem cells), as previously described [13]. The medium was centrifuged at 300×*g* for 10 min and 2000×*g* for 10 min to eliminate dead cells and cell debris. The supernatant was then centrifuged at 10,000×*g* for 30 min and 110,000×*g* for 70 min to collect exosomes in a SW32ti supercentrifuge rotor (Beckman L-100, Beckman Coulter, Brea, CA, USA). The pellets were resuspended in 2 mL phosphate-buffered saline (PBS) and re-ultracentrifuged in a SW60ti supercentrifuge rotor at 110,000×*g* for 70 min. The pelleted exosomes were resuspended in 200 μL PBS and stored at − 80 °C or used for subsequent experiments. All procedures were conducted at 4 °C.

### Identification of exosomes

#### Nanoparticle tracking analysis (NTA) of size distribution

After isolation, exosomes were diluted into 700 μL sterile PBS and evenly mixed. A NanoSight LM 10 instrument (Malvern Panalytical, Malvern, UK) was used to estimate the size distribution of the CD34^+^-Exos and miR-26a-CD34^+^-Exos.

#### Transmission electron microscopy (TEM) for exosome morphology

The morphology of the CD34^+^-Exos and miR-26a-CD34^+^-Exos was observed using TEM. Briefly, 7 μL of exosome suspension was pipetted onto a hydrophilized copper mesh for 5 min. Exos were then stained with 2% uranyl acetate for 1 min. After drying, the morphology of Exos was examined using TEM (FEI TF 20, Philips, Amsterdam, Netherlands).

#### Western blotting for exosome-specific surface markers

The expression of the positive exosome biomarkers Alix, CD9, CD63, and CD81 and the expression of the negative biomarkers of exosomes Calnexin were examined using western blotting as previously described [31]. The protein of exosomes was extracted using a Total Exosome RNA & Protein Isolation Kit (Invitrogen, Carlsbad, CA, USA), according to the manufacturer’s specifications. The protein concentration of the exosomes was determined using the bicinchoninic acid (BCA) protein assay (Beyotime), with bovine serum albumin (BSA) as a standard. All samples were adjusted to equal protein concentrations and then diluted with 6× loading buffer and denatured at 95 °C for 5 min. Equal amounts of total protein were separated on 10% sodium dodecyl sulfate polyacrylamide gel electrophoresis (SDS-PAGE) and then transferred to a polyvinylidene difluoride membrane (Millipore, Billerica, MA, USA). Immunoblots were blocked for 4 h with 5% non-fat dried milk in Tris-buffered saline/Tween-20 buffer (TBST) at room temperature and then incubated overnight (12–16 h) at 4 °C with the indicated primary antibodies against Alix, CD9, CD63, and CD81 (Proteintech, Chicago, IL, USA) followed by incubation with rabbit anti-pig IgG secondary antibody (1:5000 dilution; Beijing Biosynthesis Biotechnology, Beijing, China) at room temperature. The membrane was washed three times in TBST, and the results were analyzed using Quantity One one-dimensional analysis software (Bio-Rad).

#### Quantitative polymerase chain reaction (qPCR) for miR-26a expression in exosomes

An exosome RNA purification kit (EZ Bioscience, Beijing, China) was used to extract miR-26a from miR-26a-CD34^+^-Exos. The microRNA Reverse Transcription Kit PLUS (EZ Bioscience) was used to acquire cDNA of miR-26a. The 2× qPCR Mix for microRNA (ROX1 plus) (EZ Bioscience) was used for amplification of miR-26a. Relative miR-26a expression was normalized to U6. The primers (BioTNT, Shanghai, China) are listed in Table 1.

**Table 1:** The primers sequences used for qPCR

Genes	Forward primer sequence (5′–3′)	Reverse primer sequence (5′–3′)
miR-26a	CGTCCTTCAAGTAATCCAGGA	GCAGGGTCCGAGGTATTC
ALP	CAAGGATGCTGGGAAGTCCG	CTCTGGGCGCATCTCATTGT
RUNX2	CCGAGACCAACCGAGTCATTTA	AAGAGGCTGTTTGACGCCAT
COL I	GGAGAGTACTGGATCGACCCTAAC	CTGACCTGTCTCCATGTTGCA
GAPDH	GAAGGTGAAGGTCGGAGTC	GAAGATGGTGATGGGATTTC

### Cell counting kits for proliferation assay

Cell counting kits (CCK-8, Dojindo, Kumamoto, Japan) were also used to test cell proliferation, to estimate the proliferation of BMSCs and HUVECs in different culture situations. We seeded 5000 cells per well of one 96-well plate and cultured cells with the following different treatments: saline, 10 μM dexamethasone (DEX; Solarbio, Beijing, China), DEX+CD34^+^-Exos, DEX+miR-26a-CD34^+^-Exos, CD34^+^-Exos, or miR-26a-CD34^+^-Exos (50 μg/mL). OD values were measured on days 0, 1, 3, 5, and 7 of culture.

### Uptake of exosomes

Uptake of exosomes was observed by fluorescence. First, CD34^+^-Exos and miR-26a-CD34^+^-Exos were labeled with PKH26 red dye (Sigma Aldrich, St Louis, MO, USA) according to the manufacturer’s protocol. Then, exosomes were ultracentrifuged at 110,000×*g* for 70 min and the supernatant was discarded. The pellet was resuspended in 500 μL dilution C, and 4 μL PKH26 dye was dissolved in another 500 μL dilution C. The two tubes of dilution C were mixed and co-incubated for 5 min at room temperature to obtain exosomes-PKH26. The exosomes-PKH26 were then co-cultured with BMSCs and HUVECs at a concentration of 20 μg/mL. After 12 h, cells were fixed with 4% paraformaldehyde (Well, Shanghai, China) for 15 min, treated with 0.1% Triton X-100 (Beyotime) for 5 min for membrane penetration, stained with DAPI for 5 min, and washed with PBS three times. Photographic images were acquired using a fluorescence microscope (Olympus IX 70).

### Effects of miR-26a-CD34^+^-Exos on HUVECs in vitro

#### Tube formation assay

HUVECs were seeded into a six-well plate and treated with 10 μM DEX, CD34^+^-Exos, or miR-26a-CD34^+^-Exos (50 μg/mL) in serum-free medium. Matrigel (Corning, New York, USA) was dissolved in a refrigerator at 4 °C overnight in advance, and the required 24-well plates and 200-μL tips were placed in a − 20 °C freezer. The next day, an ice box was placed on a clean bench with a 24-well plate on ice. The Matrigel gel was quickly pipetted into a 24-well plate (200 μL per well). The plate was transferred to the cell culture incubator for 30 min for the Matrigel to solidify. The cells were digested, centrifuged at 1000 rpm for 5 min, and the supernatant was removed. The cells were resuspended in serum-free medium and counted. Each group of 2.5 × 10^4^ cells was seeded in a 24-well plate. After 8 h, the plate was observed under a light microscope (Olympus IX 70) and photographed.

#### Transwell migration assay

HUVECs were preconditioned as aforementioned. Approximately 2 × 10^4^ cells were plated into the upper chambers of a transwell plate (Corning). Complete culture medium, placed in the lower chamber, was used as a chemoattractant. Twenty-four hours later, the membranes were fixed with ethanol and stained with crystal violet (Beyotime). Then, the membranes were mounted and observed under a light microscope (Olympus IX 70).

#### Scratching experiment

HUVECs were seeded into a six-well plate. After the cells were 90–100% confluent, the complete medium was replaced with serum-free medium. A 200-μL pipette tip was used to create a scratch in the cell layer. The cells were washed three times with PBS to remove cell debris and other foreign bodies. Serum-free medium or serum-free medium containing 10 μM DEX, 50 μg/mL CD34^+^-Exos, or miR-26a-CD34^+^-Exos was added to the appropriate wells of a six-well plate. At 0, 12, or 24 h after scratching, pictures were taken under the light microscope (Olympus IX 70).

### Effects of miR-26a-CD34^+^-Exos on BMSCs in vitro

#### Alizarin red staining and alkaline phosphatase (ALP) activity

After cell density reached 60–70%, the cells were changed to osteogenic differentiation medium (Cyagen, Guangzhou, China) to promote differentiation. Then 10 μM DEX, CD34^+^-Exos (50 μg/mL), or miR-26a-CD34^+^-Exos (50 μg/mL) were added to the BMSC cultures. Alizarin red staining (Cyagen) was performed on day 21 after incubation. Cells were washed three times in PBS and fixed in 4% paraformaldehyde for 15 min. Alizarin red staining was used to determine osteogenic activity. ALP activity was evaluated using a BCIP/NBT Alkaline Phosphatase Color Development Kit (Solarbio, Beijing, China) on day 7 of incubation. The images were acquired using a light microscope (Olympus IX 70). After the Alizarin red staining was observed by the microscope, the stained calcium nodules were eluted by 10% cetyplyridinium chloride for 1 h, and the absorbance of the solution was read on the microplate reader at 550 nm.

#### Analysis of osteogenic-related gene expression using qPCR

An EZ-press RNA Purification Kit (EZ Bioscience, Beijing, China) was used for RNA extraction according to the manufacturer’s protocol. A Color Reverse Transcription Kit (with gDNA Remover) (EZ Bioscience) was used for RNA reverse transcription to obtain cDNA according to the manufacturer’s protocol. Then the 2× Color SYBR Green qPCR Master Mix (ROX 2 plus) (EZ Bioscience) was used for cDNA amplification analysis according to the manufacturer’s protocol. GAPDH was used as the internal reference for standardization. Primers (BioTNT, Shanghai, China) used for the amplification reaction are listed in Table 1.

### Effects of miR-26a-CD34^+^-Exos on femoral heads in a rat model

#### Animal model and grouping

Forty healthy female Sprague–Dawley (SD) rats weighting 260–280 g were used in this study. The rats were randomly and equally divided into four groups, *n* = 10 per group: (1) control group (treated with normal saline); (2) MPS group (methylprednisolone; rats with MPS-induced ONFH); (3) CD34^+^-Exos group (rats with MPS-induced ONFH; treated with CD34^+^-Exos); (4) miR-26a-CD34^+^-Exos group (rats with MPS-induced ONFH; treated with miR-26a-CD34^+^-Exos). After MPS injection, rats in the CD34^+^-Exos group and the miR-26a-CD34^+^-Exos group were administered 100 μg exosomes (suspended in 200 μL PBS), and rats in the MPS group were administered an equal volume of PBS [31].

#### Micro-CT scanning

After 6 weeks, the rats were sacrificed and the femoral heads were removed and fixed overnight in formalin. The femoral heads were analyzed using a SkyScan-1176 micro-computed tomography (μCT) system (Bruker micro-CT, Kontich, Belgium). The resolution of the scanner was 9 μm per pixel. Images in three planes (coronal section, sagittal section, and transverse section) of each femoral head were generated using DataViewer (Bruker micro-CT). Parameters of the trabecular bone including trabecular thickness (Tb.Th), trabecular separation (Tb.Sp), bone volume per tissue volume (BV/TV), and trabecular number (Tb.N) were analyzed.

#### Angiography

After anesthesia, the rats were placed on the operating table. The aorta ventralis was exposed and perfused with heparin saline and formalin for 20 min at suitable pressure. Next, micro-fil (Flow Tech, Inc., Carver, MA, USA) was injected slowly in case of capillary rupture. The rats were stored in a 4 °C refrigerator overnight after perfusion. Then the femoral heads were removed, fixed in formalin and decalcified for 1 month. Finally, the samples were scanned using a SkyScan-1176 micro-computed tomography (μCT) system (Bruker Micro-CT), and the vessel system of the femoral head was reconstructed and quantified.

#### Histological and immunohistochemical (IHC) analysis

The fixed, decalcified femoral heads were embedded in paraffin then deparaffinized in xylene, dehydrated in different concentrations of ethanol and rinsed in distilled water. Hematoxylin and eosin (H&E) staining was conducted for histological observations. Additionally, IHC analysis of COL I and vascular endothelial growth factor (VEGF) were performed to observe osteogenesis and angiogenesis, respectively. Images were acquired with a light microscope (Nikon ECLIPSE80i, Tokyo, Japan). Antibodies were obtained from Abcam (Cambridge, UK).

### Statistical analysis

All experiments were repeated at least three times. Data are presented as means ± standard deviation (SD). Comparisons between means of multiple groups were analyzed using one-way and two-way analysis of variance (ANOVA). Independent-sample *t* tests were used to compare means between two different groups. Statistical analysis was performed using SPSS 20.0. *P* values < 0.05 were considered statistically significant.

## Results

### Transfection efficiency and cell viability

**Fig. 1:**
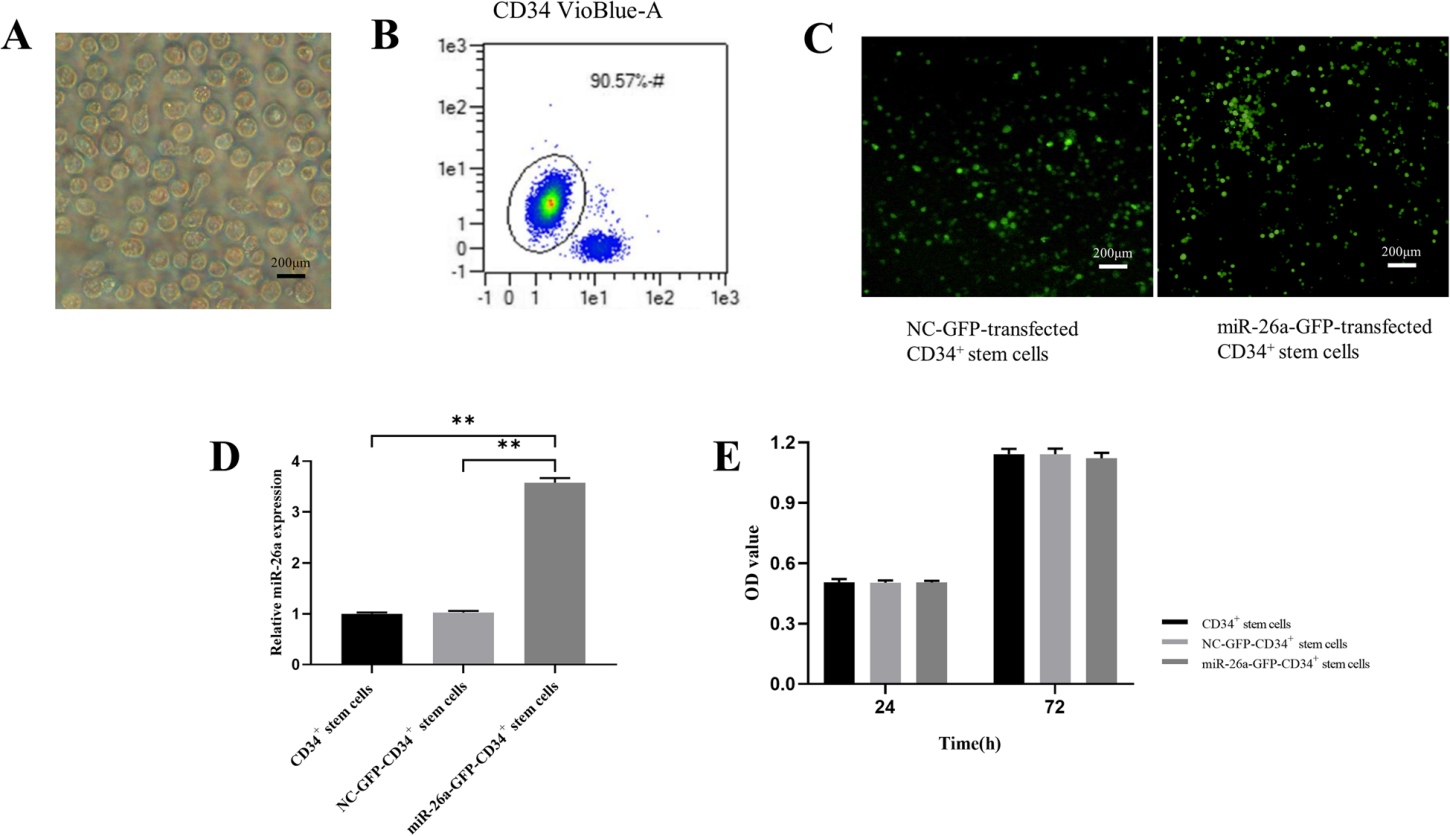
Transfection efficiency of miR-26a in CD34^+^ stem cells and cell viability. **a** Morphology of CD34^+^ stem cells shown by light microscopy. Scale bar: 200 μm. **b** Surface markers of CD34^+^ stem cells measured by flow cytometry. **c** CD34^+^ stem cells transfected with miR-26a successfully observed under a fluorescence microscope. Scale bar: 200 μm. **d** miR-26a was overexpressed in CD34^+^ stem cells compared with the non-infected cells. **e** CD34^+^ stem cell viability showed no significant differences between the non-infected CD34^+^ stem cells and the CD34^+^ stem cells infected with miR-26a. **P < 0.01

During the rapid expansion period, the primary CD34^+^ stem cells exhibited representative spherical morphology and were suspended in the culture medium (Fig. 1a). Flow cytometric analyses showed that they were highly positive for CD34 (Fig. 1b). Then CD34^+^ stem cells were transfected with lentiviral particles carrying the miR-26a gene and NC-GFP-transfected CD34^+^ stem cells and stable miR-26a-GFP-transfected CD34^+^ stem cells were observed (Fig. 1c). As depicted in Fig. 1d, miR-26a transfection in CD34^+^ stem cells had no obvious impact on cell proliferation compared with NC-GFP-transfected CD34^+^ stem cells. Finally, the stable expression of miR-26a was detected by qPCR, which showed that miR-26a-GFP-transfected CD34^+^ stem cells had significantly higher miR-26a expression compared with the NC-GFP-transfected CD34^+^ stem cells (Fig. 1e).

### Characterization of exosomes and miR-26a expression in CD34+-Exos

**Fig. 2:**
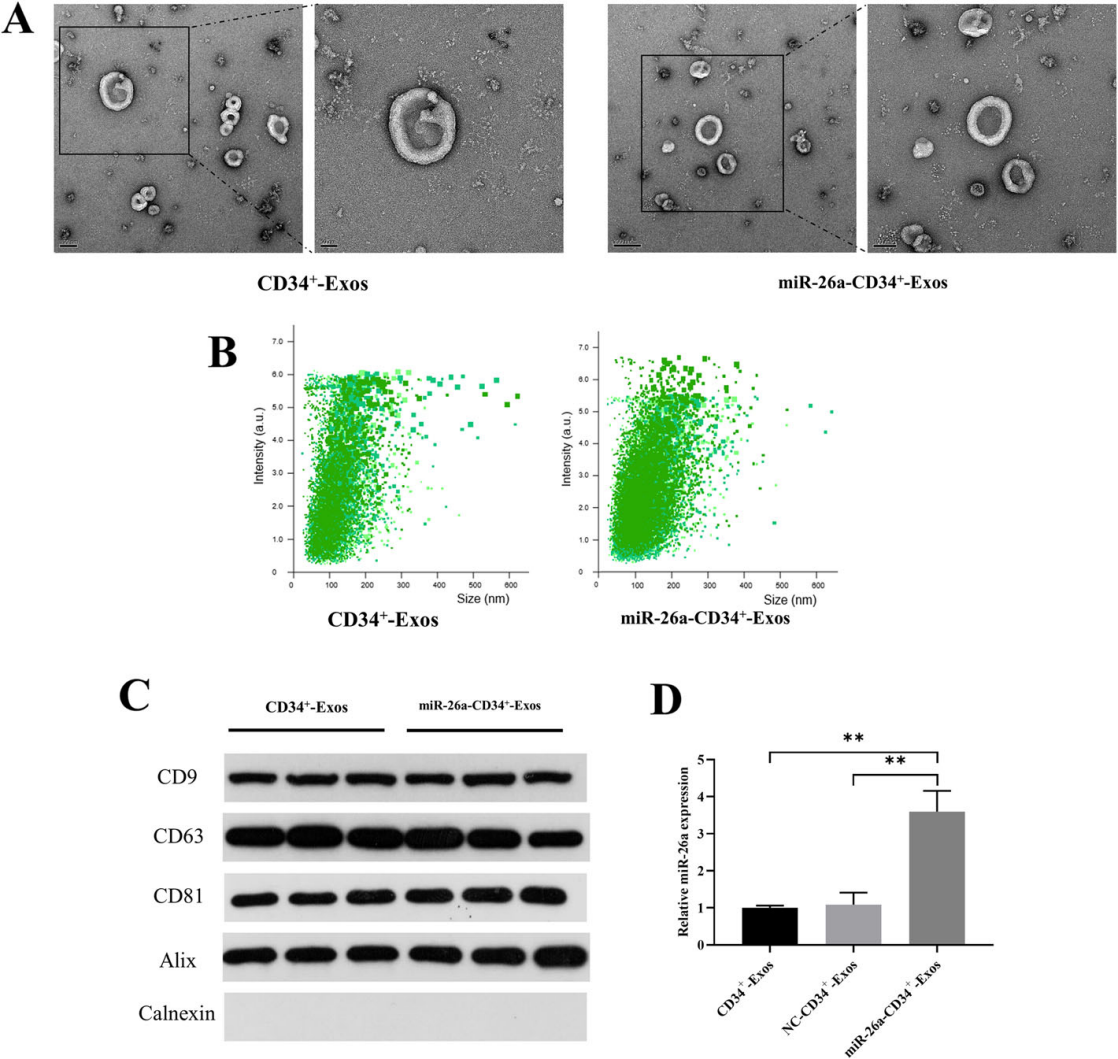
Characterization of exosomes and expression of miR-26a in CD34+-Exos. **a** Morphology of CD34^+^-Exos and miR-26a-CD34^+^-Exos observed by transmission electron microscopy (TEM). Scale bar: 50 nm. **b** Size distribution of CD34^+^-Exos and miR-26a-CD34^+^-Exos determined by nanoparticle tracking analysis (NTA). **c** Expression of the positive specific biomarkers CD9, CD63, CD81, and Alix and the negative specific biomarkers Calnexin were verified by western blotting. **d** Expression of miR-26a in miR-26a-CD34^+^-Exos was tested by qPCR. **P < 0.01

In order to characterize the isolated CD34^+^-Exos and miR-26a-CD34^+^-Exos, TEM, western blotting, and NTA analyses were carried out. TEM (Fig. 2a) showed that exosomes exhibited a spherical shape and ranged in diameter between 40 and 150 nm, which was in accordance with the nanoparticle analyses (Fig. 2b). Western blotting revealed that the surface biomarkers of exosomes including CD63, CD81, CD9, and Alix were all positive and that of Calnexin was negative (Fig. 2c). The expression of miR-26a in CD34^+^-Exos was detected by qPCR (Fig. 2d). In miR-26a-CD34^+^-Exos, miR-26a abundance was significantly higher than that in CD34^+^-Exos and NC-CD34^+^-Exos.

### miR-26a-CD34^+^-Exos increased angiogenesis and migration activity of endothelial cells in vitro

**Fig. 3:**
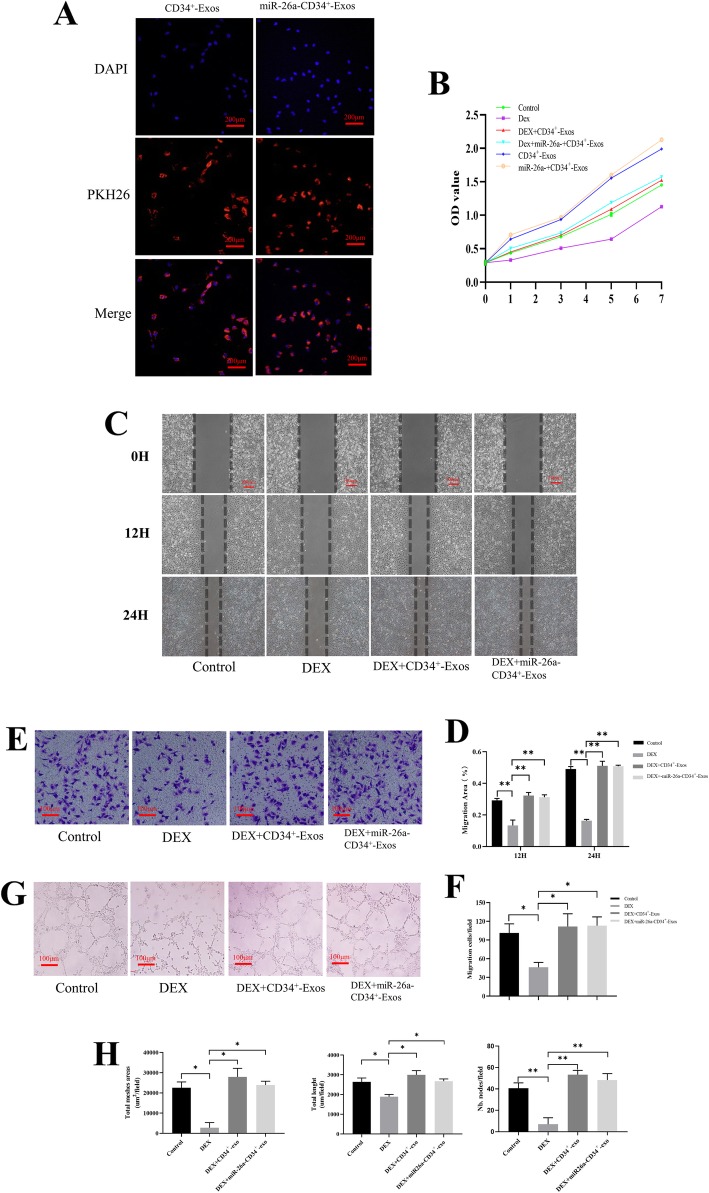
Angiogenesis was enhanced by miR-26a-CD34^+^-Exos in vitro. **a** Uptake of CD34^+^-Exos and miR-26a- CD34^+^-Exos shown by fluorescence microscopy. The PKH26 red-labeled Exos were localized in the perinuclear region of HUVECs. Scale bar: 200 μm. **b** The proliferative effects of different treatments on HUVECs were tested by CCK-8 assay. **c** Scratching experiment for evaluating the migration capacity of HUVECs at 0, 12, and 24 h in the different groups. Scale bar: 100 μm. **d** Quantitative analysis of the migration area of HUVECs. **e** The migration capacity of HUVECs was investigated by transwell assay in different groups. Scale bar: 100 μm. **f** Quantitative analysis of cell migration by transwell assay. The number of migrated cells was calculated. **g** Tube formation assay for detecting the tube-forming ability of HUVECs in the different groups. Scale bar: 100 μm. **h** Quantitative analysis of tube formation. The value of the total mesh area, total length, and number of nodes were measured. *P < 0.05, **P < 0.01

Images from the uptake experiment verified that CD34^+^-Exos and miR-26a-CD34^+^-Exos could be incorporated into HUVECs and exosomes were visible in the perinuclear region (Fig. 3a). CCK-8 assay results revealed that CD34^+^-Exos could resist the impairing effect of GCs on HUVECs and promote the proliferation of HUVECs (Fig. 3b). In the scratching experiment and transwell assay, DEX attenuated the migration ability of HUVECs compared to the control group (Fig. 3c, e). CD34^+^-Exos or miR-26a-CD34^+^-Exos rescued the migratory ability of HUVECs under the influence of DEX, as the migration areas in the scratching experiment and the number of migrated cells in the transwell assay were both significantly restored (Fig. 3d, f). In the tube formation assay, the DEX group showed a significant anti-angiogenic manifestation compared with the control group. CD34^+^-Exos and miR-26a-CD34^+^-Exos reversed the inhibitory effect of DEX on angiogenesis and increased the loop formation ability of HUVECs (Fig. 3g). The values of total mesh area, total length, and number of nodes were increased similarly after adding CD34^+^-Exos or miR-26a-CD34^+^-Exos (Fig. 3h). These results showed that CD34^+^-Exos and miR-26a-CD34^+^-Exos can promote angiogenesis and cell migration in vitro. miR-26a had no obvious influence on the angiogenic ability of CD34^+^-Exos.

### miR-26a-CD34^+^-Exos upregulate osteogenic activity in vitro

**Fig. 4:**
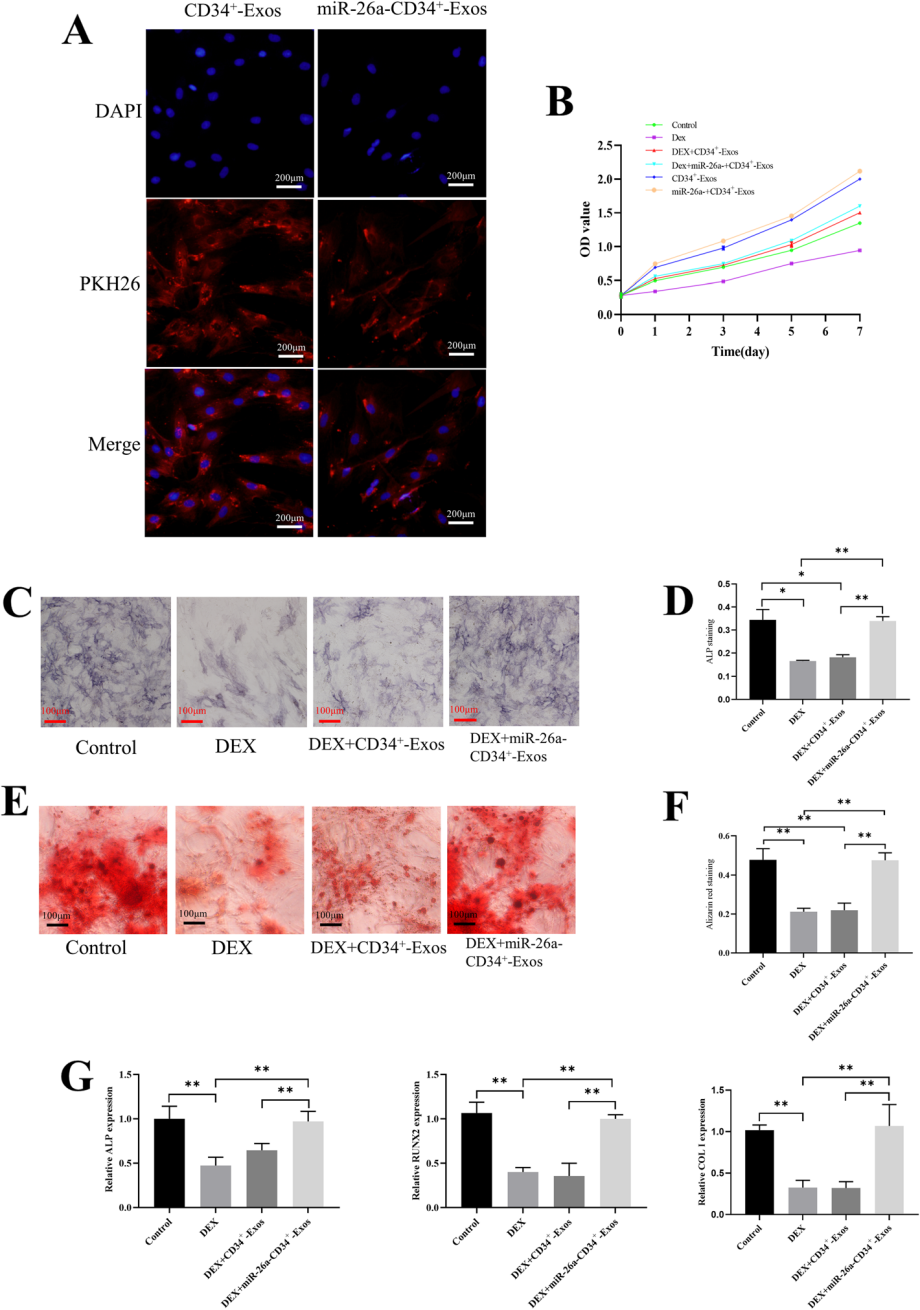
miR-26a-CD34^+^-Exos promote osteogenic activity of BMSCs in vitro. **a** Uptake of CD34^+^-Exos and miR-26a- CD34^+^-Exos shown by fluorescence microscopy. The PKH26 red-labeled Exos were localized in the perinuclear region of BMSCs. Scale bar: 200 μm. **b** The proliferative effects of different treatments on BMSCs were measured using a CCK-8 kit. **c** Expression of ALP was determined by ALP staining in different groups. Scale bar: 100 μm. **d** Quantitative analysis of ALP activity. The ratio of the area of ALP staining to total area was measured. **e** Formation of calcium nodules was analyzed by Alizarin red staining in the different groups. Scale bar: 100 μm. **f** Quantitative analysis of Alizarin red staining. The ratio of the area of Alizarin red staining to total area was measured. **g** The expression levels of osteogenesis-related genes including ALP, RUNX2, and COL I were evaluated by qPCR in the different groups. *P < 0.05, **P < 0.01

The uptake experiment showed that BMSCs could take up CD34^+^-Exos and miR-26a-CD34^+^-Exos, which accumulated in the perinuclear region of BMSCs (Fig. 4a). The proliferation rate of BMSCs under the influence of GCs was analyzed by CCK-8 assay. Results showed that miR-26a-CD34^+^-Exos could oppose the impact of GCs and increase the proliferation rate of BMSCs (Fig. 4b). To observe the osteogenic effect, ALP activity and Alizarin red staining were used to evaluate the formation of calcium nodules after induction. Results showed that miR-26a-CD34^+^-Exos reversed the osteogenic inhibition of BMSCs caused by DEX in vitro. Higher ALP activity and more calcium nodules were observed in the miR-26a-CD34^+^-Exos group than in the DEX or CD34^+^-Exos groups, and the quantified results of ALP activity and 3D calcium nodules have proved the same consequences (Fig. 4c-f). qPCR results showed that the expression of ALP, RUNX2, and COL I was downregulated in the DEX group, while only miR-26a-CD34^+^-Exos could rescue this inhibitory effect on mRNA levels (Fig. 4g).

### miR-26a-CD34^+^-Exos protect the femoral head from necrosis in a rat ONFH model

**Fig. 5:**
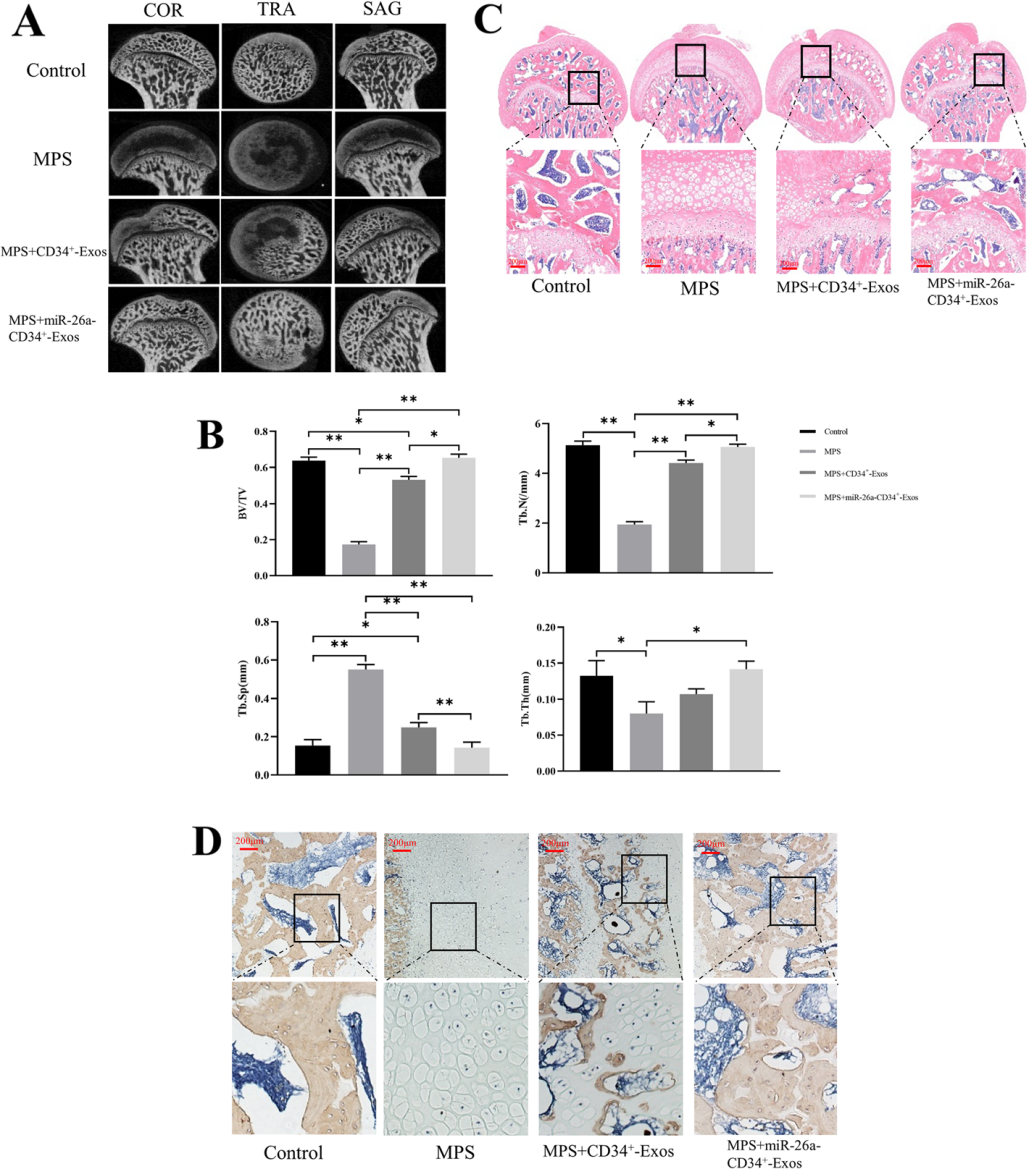
miR-26a-CD34^+^-Exos play a bone tissue-protective role in a rat model of ONFH. **a** Three plane images including coronal section (COR), sagittal section (SAG), and transverse section (TRA) of the femoral head were reconstructed in the different groups. **b** Quantitative analysis of micro-CT results. Trabecular thickness (Tb.Th), trabecular separation (Tb.Sp), bone volume per tissue volume (BV/TV), and trabecular number (Tb.N) were analyzed in the different groups. **c** HE staining of the femoral head in the different groups. Scale bar: 200 μm. **d** IHC images showing COL I staining of the femoral head in the different groups. Scale bar: 200 μm, *P < 0.05, **P < 0.01

In order to explore the effects of miR-26a-CD34^+^-Exos in vivo, we established the MPS-induced rat model of ONFH. According to the micro-CT results (Fig. 5a), greater loss of bone mineral and more cystic degeneration were observed in the subchondral area of the femoral heads in the MPS group compared with the normal rats. Moreover, the trabecular bone of the femoral head was sparse and thin. In contrast, all these deteriorations were ameliorated in the CD34^+^-Exos and the miR-26a-CD34^+^-Exos groups. Qualitative parameters such as Tb.Th, BV/TV, and Tb.N were significantly increased in the CD34^+^-Exos group and the miR-26a-CD34^+^-Exos group compared with the MPS group while the Tb.Sp was decreased (Fig. 5b), showing a rescue effect. It is worth noting that the osteogenic ability in the miR-26a-CD34^+^-Exos group was improved significantly compared to that in the CD34^+^-Exos group.

H&E staining (Fig. 5c) showed that in the MPS group, there were more empty lacunae and more pyknotic osteocytes in the cavities of the trabecular bone, and the structure of the trabecular bone was sparse. In the CD34^+^-Exos group, slight osteonecrosis of the trabecular bone was observed. In contrast, in the miR-26a-CD34^+^-Exos group, no empty lacunae or pyknosis was observed, and the trabecular bone maintained a complete structure.

**Fig. 6:**
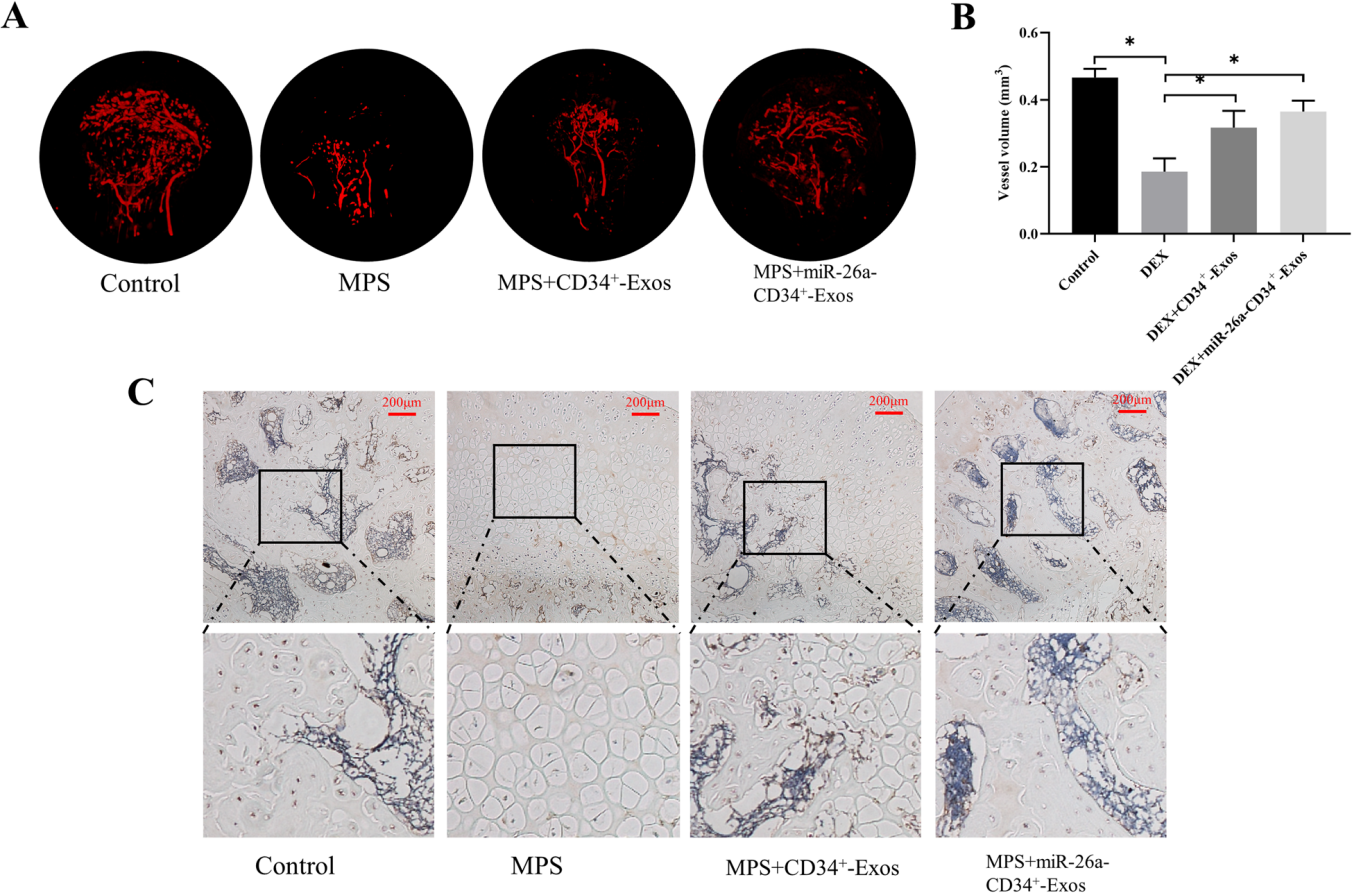
Neovascularization was enhanced by CD34^+^-Exos in a rat model of ONFH. **a** Angiography images. The blood supply of the femoral head in the different groups was measured by micro-fil perfusion. **b** The quantified result of vessel volume in the different groups. **c** IHC images showing VEGF staining of the femoral head in the different groups. Scale bar: 200 μm

The blood supply of the femoral head was measured by micro-fil perfusion. As shown in Fig. 6a, GCs not only destroyed the structure of the femoral head, but also impaired the vessel network. CD34^+^-Exos and miR-26a-CD34^+^-Exos reversed such impairment and significantly increased the vessel volume. Moreover, the quantified result of vessel volume had no obvious statistical difference in the CD34^+^-Exos group and in the miR-26a-CD34^+^-Exos group (Fig. 6b).

IHC results showed that COL I expression was downregulated in the MPS group, while it was partly rescued in the CD34^+^-Exos group and totally restored in the miR-26a-CD34^+^-Exos group (Fig. 5d). In addition, VEGF staining showed that CD34^+^-Exos or miR-26a-CD34^+^-Exos repaired the MPS-induced vascular damage (Fig. 6c).

These results showed that miR-26a-CD34^+^-Exos protect the femoral head from necrosis in our rat ONFH model by promoting osteogenesis and angiogenesis.

## Discussion

In the current study, we found that miR-26a-CD34^+^-Exos protected the femoral head from the influence of GCs by enhancing both angiogenesis and osteogenesis. Our in vitro results showed that miR-26a-CD34^+^-Exos promoted angiogenesis by HUVECs and osteogenesis by BMSCs. Further, our in vivo experiments revealed that miR-26a-CD34^+^-Exos significantly inhibited the occurrence of GC-induced ONFH.

A deficient blood supply is the main pathogenic factor of GC-induced ONFH. Increasing the blood supply of the femoral head is critical for the treatment of GC-induced ONFH. CD34^+^ stem cell therapy can successfully treat ischemia-associated diseases, such as limb and cardiac muscle ischemia, as has been demonstrated in animal studies and clinical trials [16, 19, 20]. However, autologous stem cell transplantation carries the risk of tumorigenicity and chromosomal variation. Interestingly, more and more studies have confirmed that stem cells play roles in the repair and regeneration of impaired tissue through secreting exosomes [32, 33]. Exosomes are the vital part of stem cells, which exert biological effects in ischemic diseases [34]. Moreover, CD34^+^-Exos are the central paracrine component of CD34^+^ stem cell-induced vessel growth [21]. Prabhu et al. found that CD34^+^-Exos improved the expression of pro-angiogenic mRNAs, such as VEGF, ANG1, and MMP9, in ischemic tissues [22]. Additionally, CD34^+^-Exos enhanced tube formation in vitro and neovascularization in vivo [21, 22]. Furthermore, they confirmed that the pro-angiogenic mechanism of CD34^+^-Exos is mediated by enriched miR-126 in CD34^+^-Exos [21, 22]. These research findings suggested that CD34^+^-Exos indeed have powerful angiogenic properties.

In the present study, we first used miR-26a-CD34^+^-Exos in the treatment of GC-induced ONFH. In accordance with previous reports, miR-26a-CD34^+^-Exos reversed the impairment caused by GCs in HUVECs and enhanced their proliferation and tube formation activity. In addition, the migratory ability of endothelial cells, a requirement for formation of the vascular plexus [35], was also weakened under GC treatment, a finding which was not mentioned in previous studies but was restored by miR-26a-CD34^+^-Exos treatment. In our GC-induced ONFH rat model, vascular density and expression of VEGF were also significantly improved by treatment in the CD34^+^-Exos and miR-26a-CD34^+^-Exos group compared to the MPS group. These results convey that CD34^+^-Exos can act as an efficient therapeutic method for GC-induced ONFH.

Due to the decreased osteogenic capacity, a feasible way to improve the osteogenic potential of CD34^+^-Exos is by increasing the proportion of osteogenic miRNA in CD34^+^-Exos for the treatment of GC-induced ONFH in our study. MiR-26a is one of the most important regulators of osteogenesis-associated anabolic activity. In past reports, scientists have found that miR-26a induces osteogenic differentiation of BMSCs and ADSCs and facilitates bone regeneration [23, 24, 36]. Moreover, miR-26a regulates the osteogenic differentiation of BMSCs and ADSCs via the Wnt and BMP signaling pathways, respectively [37]. Consistent with our findings, miR-26a has been shown to have pro-osteogenic ability under the negative impact of glucocorticoids in vitro and in vivo*.* Thus, it is a favorable way to improve the abundance of miR-26a in a local region of the femoral head.

Interestingly, miR-26a also exerted an influence on angiogenesis in some researches. Some researchers have shown that miR-26a could inhibit the activity of angiogenesis in liver cancer, acute myocardial infarction, and diabetes by directly targeting signaling pathways, such as hepatocyte growth factor-cMet pathway and BMP/Smad1 pathway [27, 28, 38]. However, other researchers also have reported that miR-26a could upregulate the expression of HIF-1α, VEGF, and Ang1 to enhance the angiogenesis in glioma and bone defect [29, 30]. Thus, it can be known that the effect of miR-26a on angiogenesis may be disease specific to some extent.

In our study, we found that miR-26a had no significant effect on angiogenesis in vivo and in vitro experiments. Although the detailed mechanism of miR-26a on angiogenesis remains unclear in GC-induced ONFH, an accessible paradigm from other studies suggested that the relative expression of protein miRNAs regulated may decide the miRNA-mediated effects. In support of this view, previous researches indicated that the overexpression of miR-26a in BMSCs would upregulate the expression of VEGF and Ang1 [29], but the transfection of miR-26a inhibitor in HUVECs also ameliorated the impaired angiogenesis by Smad1 signaling pathway [38]. Nevertheless, the main role of angiogenesis was CD34^+^-Exos in the present study. Researchers revealed that CD34^+^-Exos had strong angiogenic ability in which highly expressed miR-126 was the primary pro-angiogenic factors [21, 22]. And miR-126 could enhance angiogenesis in response to angiogenic growth factors by repressing negative regulators [39]. Therefore, we suppose that the main reason that miR-26a has no significant effect on angiogenesis is CD34^+^-Exos restrain the impact of miR-26a on angiogenesis. Certainly, it is necessary to further explore the detailed mechanism of miR-26a on angiogenesis.

Although significant progress has been made in understanding miRNAs, the application of miRNAs remains full of complexities. The membranes of exosomes show a high degree of similarity to their parent cells [14], stabilizing miRNAs within the exosome membranes and providing an efficient cell-target delivery system [40]. Therefore, we transfected lentiviral miR-26a particles into CD34^+^ stem cells to improve the osteogenic potential of CD34^+^-Exos. Under GC treatment, we found that miR-26a-CD34^+^-Exos strengthened the osteogenic differentiation of BMSCs in vitro, consistent with former research. Furthermore, degraded trabecular bone was rescued by miR-26a-CD34^+^-Exos in a GC-induced ONFH rat model. In addition, micro-CT and IHC results indicated that bone remodeling was partially restored in the CD34^+^-Exos group, which further verified the coupled relationship between angiogenesis and osteogenesis [41]. In brief, we have demonstrated for the first time that miR-26a can restore the weakened osteogenic activity in GC-induced ONFH. CD34^+^-Exos-mediated delivery of miR-26a may provide a promising therapeutic strategy to prevent GC-induced ONFH by promoting both angiogenesis and osteogenesis.

## Conclusions

In summary, our study demonstrated for the first time that miR-26a-CD34^+^-Exos exerted a curative effect against early GC-induced ONFH, both in vitro and in vivo. The pro-angiogenic effect of CD34^+^-Exos and the pro-osteogenic effect of miR-26a were combined in miR-26a-CD34^+^-Exos and functioned cooperatively in reversing the pathogenic process of GC-induced ONFH.

## Data Availability

All data used and analyzed during the current study are available from the corresponding author on reasonable request.

## Abbreviations

ADSCs: Adipose-derived stem cells
ALP: Alkaline phosphatase
BCA: Bicinchoninic acid
BMP-2: Bone morphogenetic protein-2
BMSCs: Bone marrow stromal cells
BSA: Bovine serum albumin
BV/TV: Bone volume per tissue volume
CCK-8: Cell counting kits
CD34^+^-Exos: CD34^+^ stem cell-derived exosomes
COL I: Collagen I
DEX: Dexamethasone
Exos: Exosomes
FBS: Fetal bovine serum
GC: Glucocorticoid
GFP: Green fluorescent protein
HUVECs: Human umbilical vein endothelial cells
IHC: Immunohistochemical
miR-26a-CD34^+^-Exos: miR-26a was overexpressed in CD34^+^-Exos
MPS: Methylprednisolone
NC: Negative control
NTA: Nanoparticle tracking analysis
OCN: Osteocalcin
OD: Optical density
ONFH: Osteonecrosis of the femoral head
PBS: Phosphate-buffered saline
qPCR: Quantitative polymerase chain reaction
RUNX2: Runt-related transcription factor 2
SDS-PAGE: Sodium dodecyl sulfate polyacrylamide gel electrophoresis
Tb.N: Trabecular number
Tb.Sp: Trabecular separation
Tb.Th: Trabecular thickness
TBST: Tris-buffered saline/Tween-20 buffer
TEM: Transmission electron microscopy
VEGF: Vascular endothelial growth factor
